# Duplication of the fallopian tube

**DOI:** 10.4103/0974-1208.38971

**Published:** 2008

**Authors:** Narayanan R, Rajeev MA

**Affiliations:** Departments of Obstetrics and Gynecology, Sanjeevani Hospital and Maternity Home, Kovai Road, Annur, Coimbatore, Tamil Nadu, India

**Keywords:** Congenital, fallopian tube, hysterosalpingography, infertility, mullerian duct anomaly, partial duplication, tubal pregnancy

## Abstract

Hysterosalpingography accurately delineates the uterine and tubal lumen, and hence is routinely performed for the evaluation of infertility.We observed a case of infertility where uterine cavity was normal but fallopian tubes were bifurcated at the ampullary region. Mullerian duct anomalies are reported in literature, but maldevelopment of fallopian tube in isolation is rare. This abnormality can present as infertility, ectopic pregnancy, in association with urinary tract anomalies or as failure of sterilisation method.

Anatomy of the female reproductive tract is to be outlined properly in the evaluation of infertility and more importantly in sterilisation procedures for medico-legal reasons. It provides the most accurate outline of uterine cavity and gives images of the lumen of fallopian tube.[1] We report a case where hysterosalpingogram revealed a rarely observed anomaly.

## CASE HISTORY

A 25-year-old woman reported to the hospital with inability to conceive even after 6 years of marriage. She has no previous history of conception. She is not tuberculous, diabetic or hypertensive. She gives history of having had left lower ureteric calculus which was cured with hydration and diuretics and analgesics. She does not have any cardiac, renal or endocrine disturbances. There is no history of pelvic inflammatory diseases or intrauterine contraceptive device implantation. The patient is not obese. She has well-developed secondary sexual characters. Gynaecological examination revealed no obvious abnormalities. Her haematological investigation reports were within normal limits (Haemoglobin 11 g percentage, total count 8400 cell per cubic millimetre, differential count showed polymorph 56%, lymphocytes 42% and eosinophils 2%, erythrocyte sedimentation rate 12 mm in the first hour).

**Figure 1:**
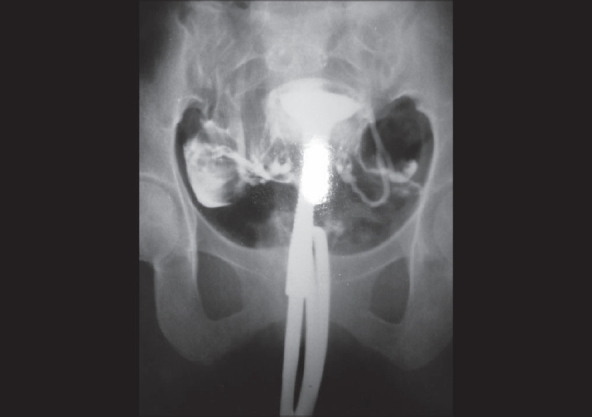
A 25-year-old woman with history of infertility whose hysterosalpingogram showed partial duplication of left fallopian tube

An ultrasound scan of the abdomen and pelvis revealed no anomaly. The procedure was done using Colvin's cannula for pushing the dye, Urograffin (Urograffin was manufactured and marketed by German Remedies and contains sodium and meglumine diatrizoate 60%, which has iodine content 292 mg/mL). Two films were taken: one after pushing 3 mL and the other after 7 mL of the dye. The film showed the uterine cavity contour to be normal without any filling defects. The fallopian tubes on both sides were visualised. The left fallopian tube bifurcated at the ampullary region, with free spillage of dye at both ends of the split tubes [Figure 1]. The right tube was visualised to be normal and patent.

After the procedure, the lady was prescribed ampicillin and metronidazole. Intravenous pyelogram and skeletal survey were done afterwards and they did not reveal any other abnormalities.

## DISCUSSION

Mullerian duct anomalies are described,[2] commonly but maldevelopment of fallopian tube in isolation is rare.[3] In the case described, we report unilateral duplication of fallopian tube with normal looking uterus and fallopian tube on the opposite side. This type of anomaly was thought to be the cause of infertility in our patient. The incidental finding of tubal duplication in HSG in our case report was reported to highlight the potential anatomical variations of fallopian tubes especially in this era of litigation.It may present as tubal pregnancy or as infertility as in our patient.It was also to emphasise as a potential reason of failure of sterilisation procedure.[4]

The importance of this documentation is to be noted in this era of litigation when one of the duplicated fallopian tubes may be missed in a postpartum sterilisation procedure.

This sort of defect can present as infertility as in our case or as tubal pregnancy.

There are reports[5] of significantly increased pregnancy rate after hysterosalpingography with the use of oil-based dye compared to water soluble contrast media. We highlight the value of hysterosalpingogram in the evaluation of anatomy of female genital tract and in the investigation of infertility. After the re-emergence of this method in the evaluation of infertility in 1980s, it has still retained value even with the usage of newer imaging and interventional techniques.[1] It is useful in delineating anatomy of uterus, tubes and tubal patency. Further, it is cost-effective and done in rural clinical settings with limited access to procedures like hysteroscopy.
